# Highly sensitive electrochemical biosensor based on redox - active monolayer for detection of anti-hemagglutinin antibodies against swine-origin influenza virus H1N1 in sera of vaccinated mice

**DOI:** 10.1186/s12917-018-1668-9

**Published:** 2018-11-06

**Authors:** Edyta Mikuła, Cristiane Erdmann Silva, Edyta Kopera, Konrad Zdanowski, Jerzy Radecki, Hanna Radecka

**Affiliations:** 1 Institute of Animal Reproduction and Food Research of Polish Academy of Sciences, Tuwima 10, 10-748 Olsztyn, Poland; 2 Universidade Estadual de Ponta Grossa - UEPG, Setor de Ciências Exatas e da Terra, Departamento de Química, Av. Carlos Cavalcanti, 4748, CEP 84030-900, Ponta Grossa/ PR, Brazil; 3 Institute of Biochemistry and Biophysics of Polish Academy of Sciences, Pawińskiego 5a, 02-106 Warsaw, Poland; 4 Research and Development Center, Olimp Laboratories, Pustynia, Dębica, Poland; 5 Institute of Chemistry, University of Natural Sciences and Humanities, 3 Maja 54, 08-110 Siedlce, Poland

**Keywords:** Detection of antibodies in mice sera, Avian swine virus, Histidine-tagged hemagglutinin monomer, Electrochemical biosensor

## Abstract

**Background:**

In this work, we report an electrochemical biosensor for the detection of anti-hemagglutinin antibodies against the swine virus H1N1 present in mice sera immunized with mixture of His_6_-H1 HA in monomeric and oligomeric form. The oriented immobilization of the recombinant His-tagged hemagglutinin (His_6_-H1 HA) consists of: (i) formation of a mixed layer of 4-mercaptobutanol (MBT) and the thiol derivative of dipyrromethene (DPM); (ii) complexation of Cu (II) by DPM; (iii) immobilization of His_6_-H1 HA via coordination bonds between Cu (II) sites from DPM–Cu (II) complex and imidazole nitrogen atoms of a histidine tag; (iv) filling free spaces with bovine serum albumin. The interactions between recombinant His_6_- H1 HA covalently attached to the electrode surface and the anti-hemagglutinin H1 antibodies present in mice sera were explored with Osteryoung square-wave voltammetry.

**Results:**

This analytical device was able to detect the antibodies present in vaccinated mice sera diluted from 1 × 10^9^ to 1 × 10^8^ fold.

**Conclusions:**

The unprecedented sensitivity of described biosensor is much better than widely use ELISA test and other analytical methods for determination of antibodies against the influenza A viruses. It has been proved that redox active DPM-Cu (II) monolayer is a universal platform suitable for stable and oriented immobilization of any His-tagged sensing elements. Thus, this universal layer could be a base of numerous analytical devices suitable for detection of antibodies against different viruses.

## Background

Influenza viruses induce annual epidemics and casual pandemics that have claimed the lives of millions. Their very high seasonal variation makes effective vaccination and environment control very challenging [1–5]. The twenty-first century pandemic resulted from an influenza A (H1N1) virus that quickly spread worldwide and was reported in 214 countries in various states, claiming many victims [6–8]. In April 2009, a pandemic H1N1 influenza virus emerged, rapidly spread and resulted in launching the pandemic scheme worldwide [4, 9–11]. The virus was circulating in the pig population prior to its transmission to humans [12, 13]. The recent study showed that dogs may play critical roles in influenza virus transmission to human [3]. These animals living so close to people may generate potential public health risk. H1N1 virus is special for its high transmission speed, although its lethality and virulence are temperate. After the WHO statement of the post-pandemic period since August 10, 2010, the influenza А (H1N1) virus continued to circulate as a casual virus [14].

There are many cases to emergence of swine-origin H1N1 viruses that have transmitted to and prevalence among humans, subsequent in outbreaks internationally [15, 16]. However, transmission of the virus from pigs to humans does not always ultimately cause flu, sometimes results only in the creation of antibodies in the blood [17]. Record of these outbreaks and real-time monitoring of the evolution of this virus are important for the infectious disease control programs and for better understanding of the factors that specify viral pathogenicity and transmissibility.

In swine, fatality is usually low (around 1–4%), but the virus can cause weight loss and poor growth, leading to the economic loss [18].

Vaccination helps prevent influenza morbidity and mortality. Effective vaccines induce protective immunity which is correlated with the presence of virus-specific antibodies in serum that are directed against the external coat proteins of the virion, hemagglutinin (HA) and, to a lesser extent, neuraminidase (NA). The level of HA-specific antibodies correlate with the vaccine protective efficacy [19–21].

Several methods for determination of antibodies against influenza virus are routinely used in serological analysis. Among them Hemagglutination Inhibition (HI) assay is the most frequently applied to detect antibodies that inhibit the interaction of influenza HA with receptors on red blood cells or cultured cells. It is an indirect assay in which the highest dilution of serum that prevents hemagglutination is called the HI titer of the serum [19, 20, 22]. Another common serological assay to measure the total serum antibodies against specific influenza antigen is ELISA test [23, 24]. All of these assays although routinely used possess some drawbacks. Their main disadvantages are the need of large sample volume and specialized equipment. In addition, they are not adequate for the construction of simple, disposable pocket like sensors for a wide application range.

Therefore, the development of effective influenza vaccines, as well as methods for viruses and antibodies detection are still challenging tasks for scientists.

The electrochemical biosensors are promising alternative to currently used detection systems. The low sample consumption, high sensitivity, selectivity, compatibility with modern micro-fabrication technologies and good possibility for miniaturization are the main reasons to attract the powerful interests which is confirmed by increasing number of publications [25–27].

The immobilization of either antibody or antigen on the sensor surface is a key step in the fabrication of most immunosensors.

One of the very frequently applied immobilization method is based on non-specific physical adsorption onto gold nano particles as well as carbon nanotubes [28–33]. The application of reaction between amino or carboxylic groups present in the protein structure with complementary reactive groups chemically attached onto solid support using EDC/NHS coupling ensure more stable sensing element immobilization. Unfortunately, the right orientation of sensing element on the sensor surface is not fully guarantee using EDC/NHS approach. This problem could be solved using antibodies F (ab’) fragments which could be self-assembled on the nano-gold surfaces through covalent bonds between Au and disulfide or thiol groups from hinge region of immunoglobulin. Such approach guarantee right antibodies orientation [34, 35].

The application of His-tag chemistry for immobilization bioanalytical active molecules on the surface of redox active monolayers deposited on the gold electrodes has solicited vivid interest among scientists. This method allows to keep the stable and oriented immobilization of sensing element onto solid support with maintaining their biological activity [36, 37]. In this type electrochemical biosensors, the procedure for immobilization of the specific recognition elements involved the interaction between transition metal centers forming complexes on the electrode surface and nitrogen atoms from the histidine–tag present. The formation of antigen-antibody complex at the electrode; aqueous sample interface affects the accessibility of ions from supporting electrolyte towards the redox centres embedded into sensing layers. The outcome of this phenomenon are changes of redox centres characteristics, which is the base of electrochemical signal generation [36]. The application of His-tag chemistry allows to keep the stable and oriented immobilization of sensing element onto solid support with maintaining their biological activity [37, 38]. Moreover, using this type of biosensors, the presence of electroactive markers in the sample solution is not required. This is very crucial for analytical procedures involving naturally occurring molecules, whose properties might be influenced by redox markers.

In our previously published paper [36], we have proposed the redox active layer consisted with dipyrromethene (DPM)–Cu (II) complex for stable and oriented immobilization of histidine – tagged fragment of avian influenza H5N1 virus. This sensor was suitable for selective and sensitive detection of antibodies against avian influenza virus type H5N1 present in hen sera.

Thus, we have applied the same approach in the research presented in order to evaluate immunization efficiency of his-tagged recombinant hemagglutinin derived from H1N1 used for mice vaccination. The hemagglutinin (HA) from the H1N1pdm09 influenza virus (His_6_-H1 HA) attached to the DPM-Cu (II) redox active layer deposited on gold electrode was applied as a sensing element responsible for detection antibodies present in mice sera vaccinated with mixture of His_6_-H1 HA in monomeric and oligomeric form.

## Results

### Characterization of biosensor based on redox active DPM-cu (II) monolayer

**Fig. 1:**
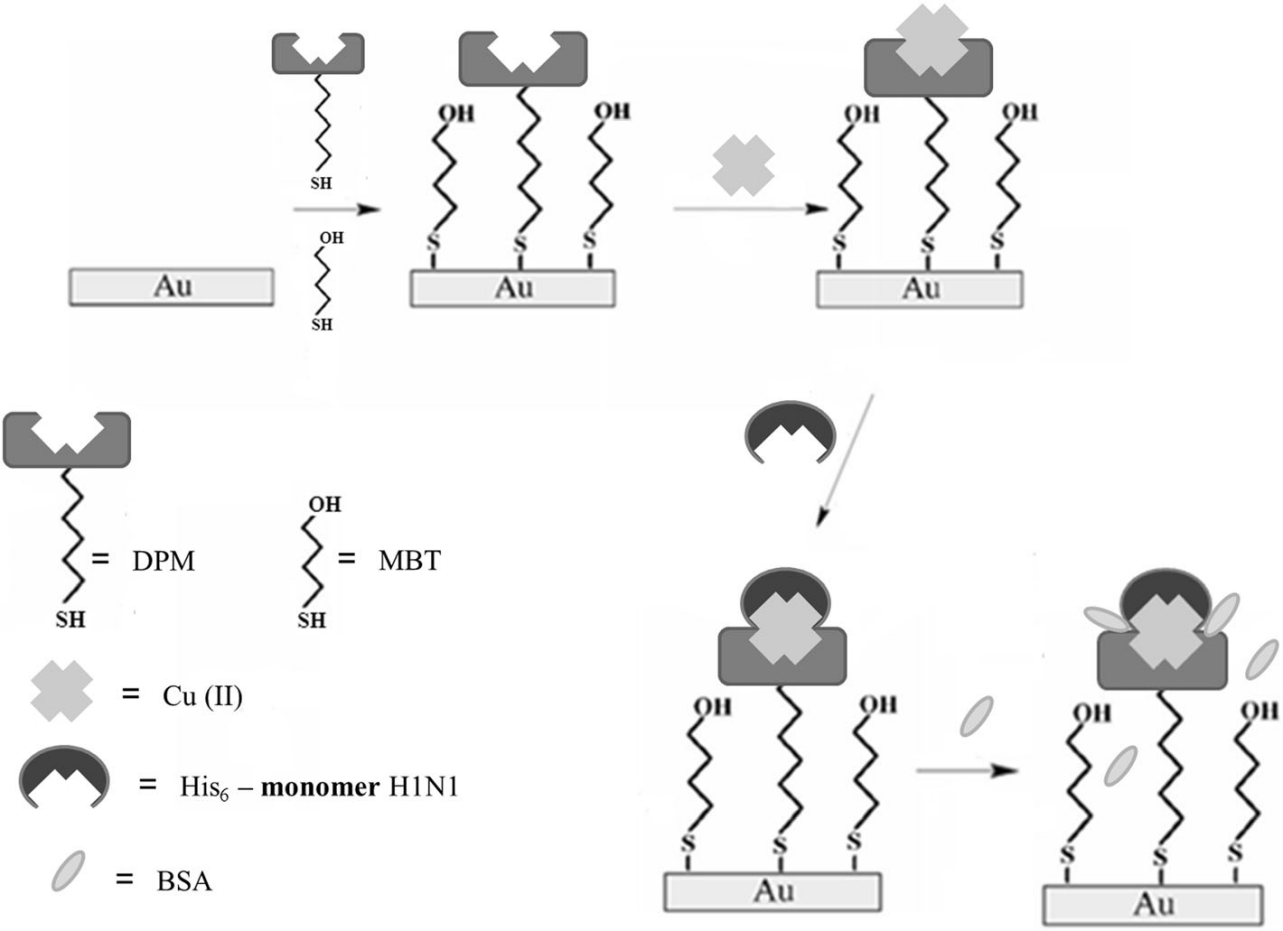
The scheme of the biosensor based on MBT/DPM–Cu (II) SAM

The process of the biosensor formation is shown in Fig. 1.

This strategy has been already applied for biosensor assembly destined for detection of antibodies against H5N1 viruses present in vaccinated hen sera [36]. Briefly, in the first step, on the Au electrode surface a mixed DPM and MBT voltammograms of a gold electrode modified by MBT. Then, Cu (II) was complexed by DPM attached to the surface of the electrode. Using such modification conditions, Cu (II) surface coverage in the range 7.35 ± 0.4 × 10^− 11^ mol cm^− 1^ was obtained [38].

Next, the recombinant histidine-tagged hemagglutinin, in monomeric form was immobilized by means of coordination bonds between Cu (II) centres and the nitrogen atoms of imidazole present in His_6_-H1 HA. This methodology allows for secure immobilization of sensing elements with keeping their right position. BSA was used to stop up any remaining free spaces on the gold electrode surface in order to abolish undefined binding.

**Fig. 2:**
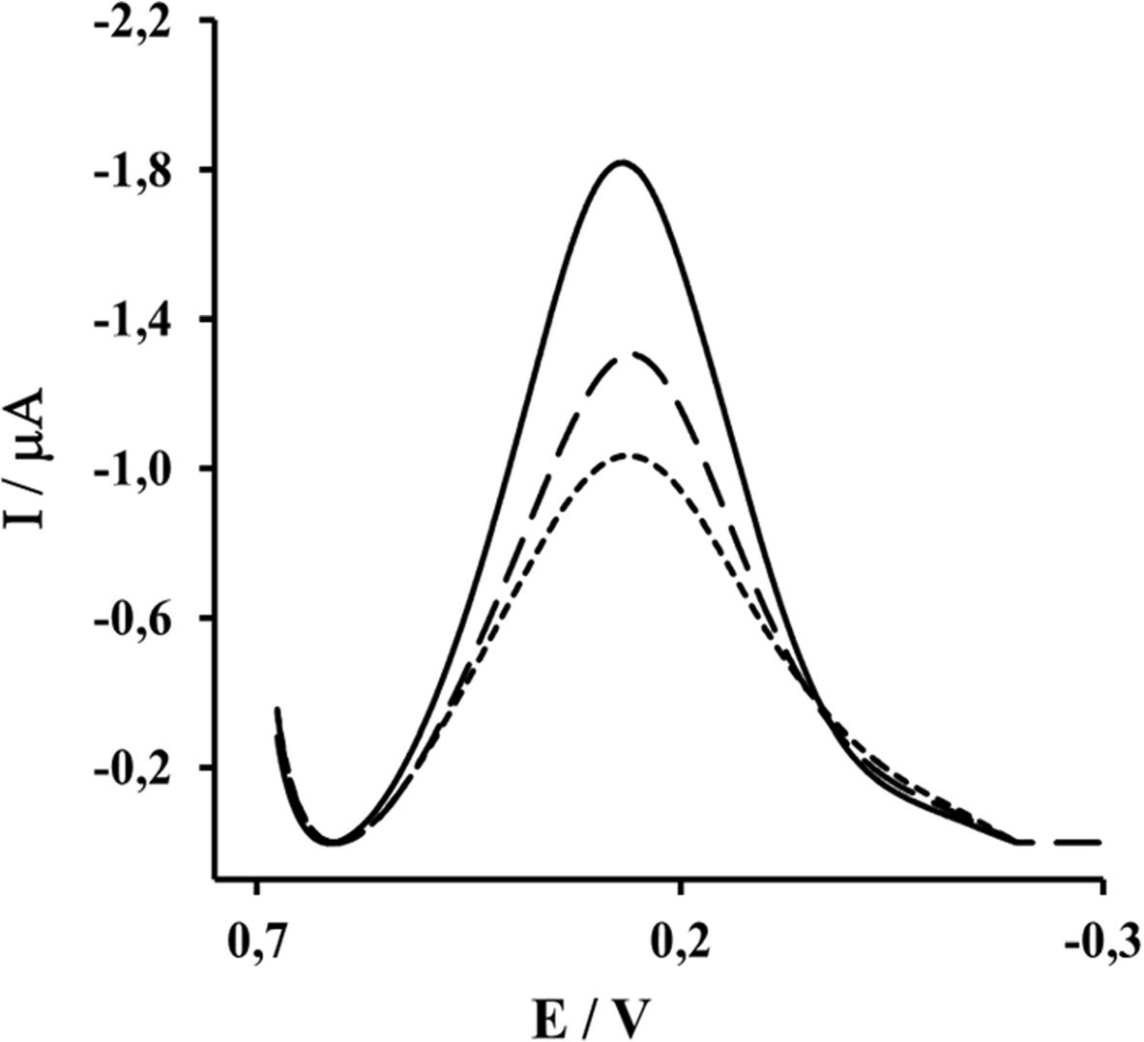
Square wave voltammograms of a gold electrode modified by MBT/DPM–Cu (II) (solid line), after immobilization of His_6_-H1 HA monomer (dashed lines); and after filling free spaces with bovine serum albumin (finely dashed lines). Measurement conditions: 0.01 M PBS (pH 7.4), scan rate 100mVs^− 1^

The immobilization of His_6_-H1 HA on the surface of a DPM–Cu (II) monolayer was examined by Osteryoung square-wave voltammetry. This technique allows to quantify the difference between the oxidation and reduction peak current in order to remove the capacitive current [39]. The DPM-Cu (II) redox peak current was visible at 267 mV ± 5 mV. The complexation reaction between Cu (II) and His_6_-H1 HA-monomer generated the drop of Cu (II) redox peaks current about 34 ± 6% (Fig. 2).

### The detection of the anti-hemagglutinin antibodies in mice sera using electrodes incorporated with His_6_-H1 HA-monomer

The presented biosensor was applied for determination anti-hemagglutinin H1 monoclonal antibodies present in mice sera vaccinated with mixture of monomeric and oligomeric form H1 HA. The reproducibility of proposed sensor was estimated based on potential of peak redox Cu (II)/Cu (I) current as well as its intensity measured in buffer free of analyte. These values have been calculated for 60 gold electrodes modified with MBT/DPM-Cu (II)-His_6_-H1HA. Obtained values 251 mV ± 19 mV and 1.67 ± 0.25 μA proved the good reproducibility of proposed sensor fabrication. A properly constructed biosensors were used once to measure antibodies concentration in the series of diluted mice sera. Next, the electrodes were cleaning, modified and again used for the measurements.

**Fig. 3:**
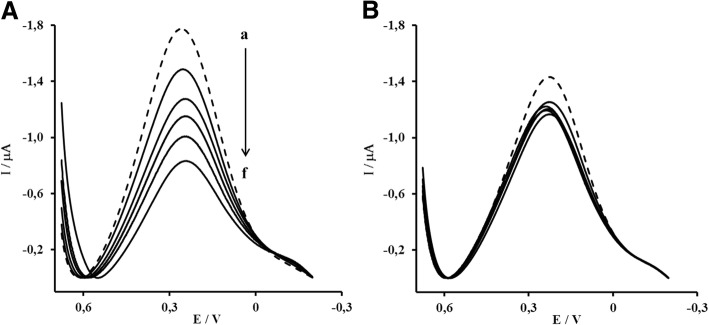
The representative OSWV responses obtained for electrodes modified with Au-MBT/DPM–Cu (II)–His_6_–H1 HA monomer in the presence of: **(a)** sera from mice after third dose 3 × 25 μg of H1HA, **(b)** sera from mice after third dose of saline and adjuvant. The sera were diluted with PBS pH 7.4 in the range from (a) 0.000 (only buffer -dashed line, (b) 1 × 10^10^, (c) 5 × 10^9^, (d) 1 × 10^9^, (e) 5 × 10^8^, (f) 1 × 10^8^ Measuring conditions: electrodes modified with: Au-MBT/DPM–Cu (II)–His_6_–H1 HA monomer, electrolyte: PBS buffer pH = 7.4 (buffer composition: 0.1368 M NaCl, 0.0027 M KCl, 0.0101 M Na_2_HPO_4_, 0.0018 M KH_2_PO_4_).

**Fig. 4:**
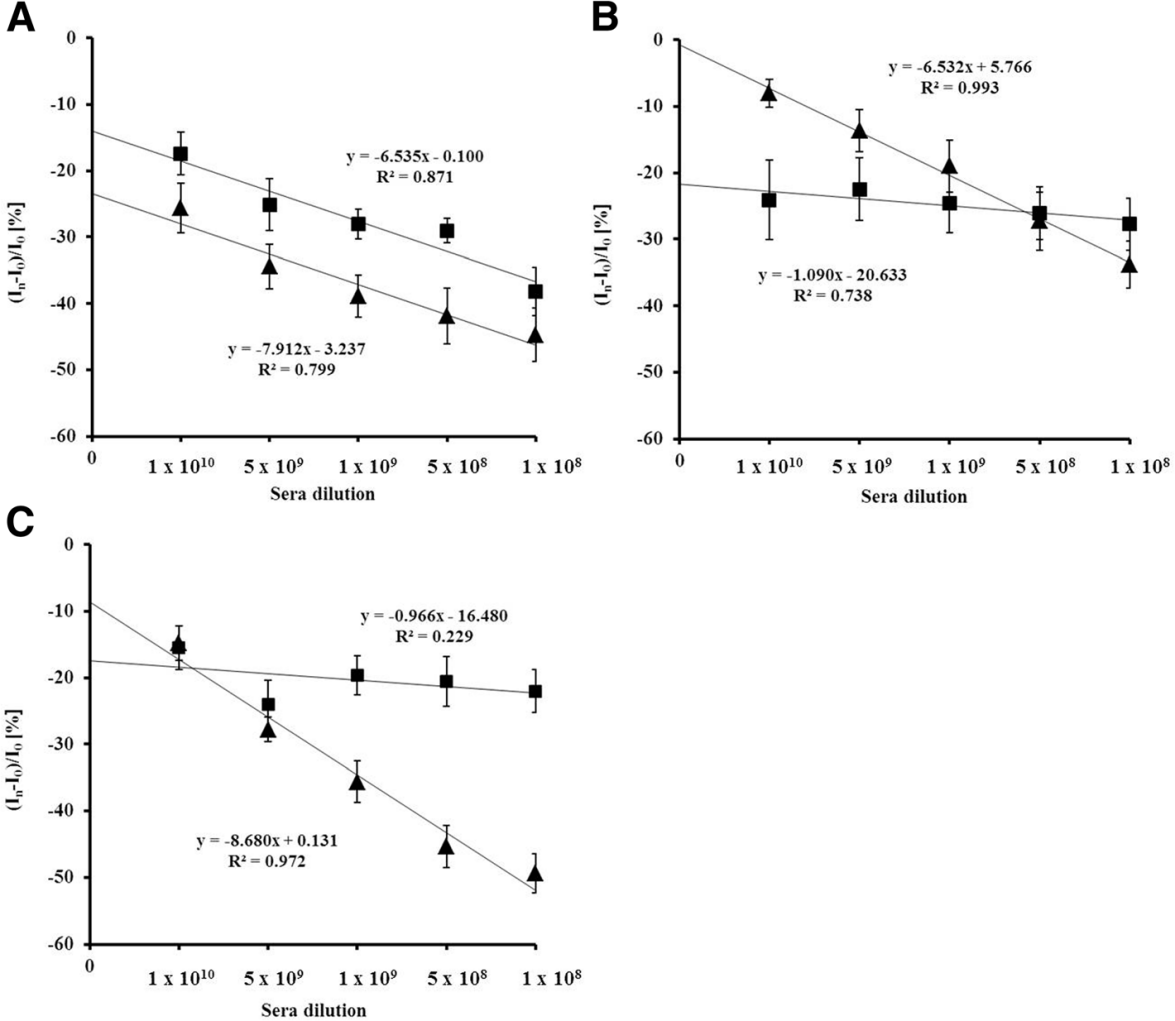
The relationship of relative Cu (II)/Cu (I) redox current decrease vs. dilution of: (▲) sera from a vaccinated mice; (■) sera from a non-vaccinated mice after: (**a**) the first dose 1 × 25 μg, (**b**) the second dose 2 × 25 μg, (**C**) the third dose 3 × 25 μg of H1 HA or saline and adjuvant mixture. Measuring conditions: see Fig. 3 caption. I_0_–Cu (II)/Cu (I) redox current measured in the presence of buffer; I_i_– Cu (II)/Cu (I) redox current measured in the presence of a particular sera dilution

The samples of blood from vaccinated mice were taken after: the first dose 1 × 25 μg of HA, the second dose 2 × 25 μg and the third dose 3 × 25 μg. The samples of blood from non-vaccinated mice, was taken at the same time interval, but the animals received only saline and adjuvant. The His_6_ -monomeric of H1 HA attached to the electrode surface (Fig. 1) was responsible for the detection of anti-H1 HA antibodies. The formation of antigen – antibody complex at the electrode surface caused a decrease of the Cu (II)/Cu (I) redox current vs. decrease of vaccinated sera dilution (Figs. 3a and 4a). The immunological response caused by monomeric form of H1 HA was selective. In sera from un-vaccinated mice the specific anti-H1 HA antibodies were not detected (Fig. 3b).

The immune-response towards His_6_-H1 HA–monomer were dose depended. After first antigen dose, the moderate responses were observed. The calibration slopes obtained for the sera taken from vaccinated and unvaccinated mice were similar (Fig. 4a). The substantial increase of antibodies level in vaccinated mice sera was observed after the third antigen dose. This was confirmed by the highest calibration slope of − 8.680 (Fig. 4c). The moderate responses, with slope of − 0.966, recorded in the presence of sera taken from mice treated only with saline and adjuvant confirm the specificity of immunization caused by monomeric form of H1 HA (Fig. 4c).

## Discussion

The proposed electrochemical biosensor allows for successful discrimination between sera of un-vaccinated and vaccinated mice against swine virus H1N1. This is evidence that immunosensor performance is not affected by matrix mice sera components, which is very crucial from analytical point of view.

The biosensor was able to detect the antibodies present in vaccinated mice sera diluted from 1 × 10^9^ to 1 × 10^8^ fold. This unprecedented sensitivity is several magnitude (10^7^) better than sensitivity widely use ELISA test for the same subtype viruses H1N1. The antibodies against influenza A/H1N1 have been detected in human and swine serum diluted only 1: 200 or 1:2000, respectively [40, 41].

The sera dilution range suitable for biosensor presented is also superior in the comparison to dilution range used for the detection of other types of viruses [36, 42–46] (Table 1).

**Table 1:** Analytical methods used for determination of antibodies against the influenza A viruses

Measurement technique	Sensing element	Analyte	Determination in real samples	References
OSWV	Recombinants hemagglutinin from the avian influenza virus type H5N1	Anti-hemagglutinin H5 monoclonal antibodies	Hen serum diluted 1:7 × 10^6^	[36]
ELISA	HA_50–274_-H1N1 in E.coli	A/H1N1/2009 antibodies	Human serum diluted 1:200 in PBS	[41]
ELISA	Influenza H1N1 X-53 antigenically identical to A/NewJersey/11/76	Antibodies against influenza A/H1N1	Swine serum diluted 1:2000 in 2% fish gelatin-tween-PBS	[42]
ELISA	Influenza H5N1 A/Chicken/HongKong/YU22 antigen	Human H5 antibodies	Human serum diluted 1:1280	[43]
EIS	Peptide chains connecting amino acid sequence 98–106 (YPYDVPDYA) of HA	HA-antibody	Not determined	[44]
ELISA	Influenza H5N1 A/Hong Kong/156/97	Antibody to avian influenza A (H5N1)	Human serum diluted 1: 409600	[45]
Western blot assay	Recombinant HA protein from A/Hong Kong/156/97	Antibody to avian influenza A (H5N1)	Human serum diluted 1: 100	[45]
Modified hemagglutination inhibition (HI) test	Horse erythrocytes and hemagglutinating units of virus type H7N7	Antibodies against the avian influenza A (H7N7) virus	Human serum diluted 1: 640	[46]
EIS	Recombinants hemagglutinin from the avian influenza virus type H5N1	Anti-hemagglutinin H5 monoclonal antibodies	Hen serum diluted 1:7 × 10^7^	[47]
OSWV	Recombinant His-tagged HA subtype H1N1- monomer from the H1N1pdm09 influenza virus	Anti-hemagglutinin antibodies against the swine virus H1N1	Mice sera diluted 1 × 10^9^–1 × 10^8^	This work

Why electrochemical immunosenors are superior in the comparison to the other transduces? There are several reasons such as: the direct conversion of binding event between antibody and antigen to an electronic signal, an excellent detection limits, small analyte volumes (in μL level), ability to be used in turbid biofluids, suitability for rapid measurements and easy miniaturization. It should be also underlined that immunosensor proposed is based on only primary antibody. The lack of the necessity of using the secondary antibody makes immunosensor fabrication simpler and cheaper in the comparison of “sandwich type” immunosensor.

Taking into account the above advantages, the electrochemical immunosensor proposed could be used as the alternative tool for widely used ELISA for control of the presence of H1N1 virus.

## Conclusions

The biosensor based on DPM-Cu (II) redox active monolayer deposited on gold electrode surface was suitable for oriented and stable immobilization of His_6_–H1 HA-monomer resposible for detection of anti-hemagglutinin antibodies against swine virus present in vacinnted mice sera.

The bisosensor was able to detect the presence of antibodies of sera sample dilluted 10^9^ fold. This remarkable sensistivity is a few order of magnitude better than widely used ELISA test. It is worst to underline, that only 10 μl sample volume is necessary to perform of analyse.

It has been proved that redox active DPM-Cu (II) monolayer is a univerasl platform suitable for stable and oriented immobilistion of any His-tagged sensing elements. Thus, this layer could be a base of numerous analytical devices suitable for detection of antibodies against different viruses.

## Methods

### Materials and chemicals

DPM was synthesized by ProChimia Surfaces Company (Sopot, Poland). 4-Mercapto-1-butanol (MBT), phosphate buffer saline (PBS) components (137 mM NaCl, 2.7 mM KCl, 1.8 mM Na_2_HPO_4_, 10 mM KH_2_PO_4_), copper (II) acetate, and chloroform were obtained from Sigma-Aldrich (Poznań, Poland). Alumina 0.3 and 0.05 μm were purchased from Buehler (Lake Bluff, IL, USA). Sulphuric acid, potassium hydroxide, and methanol were supplied by POCh (Gliwice, Poland). Bovine serum albumin (BSA) was purchased from Invitrogen Life Technologies (Darmstadt, Germany).

The recombinant H1 HA antigen (mixture of monomer and oligomer) used in this study was based on the sequence of the H1N1pdm09 (A/H1N1/Gdańsk/036/2009) and produced in *Pichia pastoris*. It contains a His_6_-tag at the N terminus. Plasmid pPICZαA (Invitrogen) and plasmid pJET/H1 carrying H1N1 virus hemagglutinin gene (A/H1N1/Gdansk/036/2009, kindly gifted by prof. Bogusław Szewczyk, UG, Poland) have been used for construction of expression vector pPICZαA/H1. *Pichia pastoris* cells of strains KM 71 (his4, aox1:ARG4, arg4) (Invitrogen) were transformed with recombinant plasmids by electroporation. The presence of recombinant protein both in medium and cells (control), was detected by SDS-PAGE and Western blotting. The H1 protein (with His-tag at the N-terminus) was purified was purified by affinity chromatography using Ni-NTA agarose (Qiagen, Germany), and its purity was analysed using 4–12% SDS-PAGE (Bio-Rad, Poland). After dialysis against PBS, pH 7.4, the protein was lyophilized and stored at − 20 **°**C. Oligomeric status of H1 HA protein was analysed on a Superdex-200 10/300 GL column (GE Healthcare, Poland), pre-equilibrated with 10 mM Tris pH 7.8 with 200 mM NaCl. Molecular weight marker standards (Bio-Rad, Poland) were used for column calibration providing the generation of standard curves to identify the molecular weights of the protein forms present in samples. The protein elution was monitored at 280 nm.

All aqueous solutions were prepared using Milli-Q water, with resistivity 18.2 MΩ·cm (Millipore, Darmstadt, Germany). Reagents and solvents were of analytical grade and used without further purification. Experiments were carried out at room temperature unless stated otherwise.

### Immunization procedure

Healthy adult female mice BALB/c (6–7 weeks old) were provided from the Animal House of Mossakowski Medical Center, Polish Academy of Sciences. The mice were housed in the individually ventilated cage (IVC) systems with controlled humidity of 55+/− 2%, a temperature of 22+/− 20C and 12-h light/dark cycle. All mice were allowed free access to food and water. After acclimatization mice were used for generating the experimental procedures. The experimental protocol was approved by the Local Ethics Committee. Procedures were adhered to guidelines published in the European directive 2010/63/UE on the protection of animals used for scientific purposes.

Seven-week old, pathogen-free, female Balb/c mice were used for vaccination. The experimental groups consisted of 10 mice. The control group was administered adjuvant only. The recombinant antigen suspended in saline solution supplemented with Alhydrogel (aluminum hydroxide) was administered subcutaneously (sc) into the neck skin fold at the dose of 25 μg. There were three injections (first application of antigen and/or adjuvant + two booster shots) at an interval of three weeks between each dose in order to monitor immunological response. Control mice received only a specific adjuvant. Blood samples were taken two weeks after each injection in order to determine the level of antibodies. Sera samples, taken from individual groups at each time point of experiment were pooled and (serially) diluted in 0.01 M PBS buffer (taking into account a molar concentration of phosphate ions). Sera were stored at − 20 °C.

All the animals were euthanized at the end of procedures in a chamber with carbon dioxide (BIOSCAPE, CO_2_ – box model L). The following ratios of CO_2_/O_2_% vol/min were applied for induction 5/95% vol/min and for eutanasia 100/0% vol/min. BIOSCAPE, CO_2_ – box model L2 meets all established recommendations and statutory provisions for euthanasia of small rodents using CO_2_ according to Annex IV of European directive 2010/63/UE, a secondary physical method of euthanasia was cervical dislocation. Euthanasia in the chamber BIOSCAPE CO_2_ model

L2 does not require earlier anaesthesia because mice are anaesthetised in the chamber during the first part of euthanasia process.

### Preparation of biosensor—Successive steps of gold electrode modification

The biosensors were prepared according to procedure previously reported [36]. Briefly, clean gold electrodes were immersed in mixed solution of 10^− 5^ M of DPM and 10^− 3^ M of 4-mercapto-1-butanol (MBT) in dichloromethane at 4 °C for 3 h. Next, after washing with dichloromethane and dichloromethane-methanol solution (1:1), the electrodes were dipped in 1 mM copper (II) acetate solution in dichloromethane-methanol mixture (1,1) for 1 h. The tubes containing electrodes were sealed with teflon tape to avoid solvent evaporation. Then, the electrodes were washing in dichloromethane-methanol solution, next methanol and in the end TRIS buffer. Next, 10 μL of 10 μg/ml His_6_- H1 HA in monomeric form dissolved TRIS buffer (10 mM, pH 7.6) were placed on the gold surfaces for 2 h. After that the electrodes were washing with TRIS buffer. Next, 10-μl droplets of BSA solution in a concentration of 1% (m/v) were applied on the surface of each electrodes and left for 30 min. After deposition of the BSA, the electrodes were washed and stored at 4 °C in TRIS buffer, pH 7,6 until used. Next, 10 μL aliquots of the respective dilutions of the mouse sera (10-fold serial dilutions in the range from 1 × 10^9^ to 1 × 10^8^) in 0.01 M PBS buffer were deposited onto the electrode surface for 30 min. The electrodes were covered by Eppendorf tubes in order to protect against evaporation and air pollution. After 30 min. of incubation at room temperature, the electrodes were rinsed with 3 mL of 0.01 M PBS, pH 7.4 in order to remove the unbound antibodies.

### Osteryoung Square wave voltammetry (OSWV) measurements

All Osteryoung Square Wave Voltammetry (OSWV) measurements were performed with a potentiostat–galvanostat AutoLab (Eco Chemie, Utrecht, The Netherlands) as stated by procedure already published [47]. Briefly, the following electrodes were used: a gold working electrode, an Ag/AgCl reference electrode and a Pt counter electrode. The potential was scanning from + 0.7 V to − 0.3 V, with a step potential of 0.001 V, a square-wave frequency of 25 Hz, and amplitude of 0.05 V. PBS buffer with the following composition: 0.1368 M NaCl, 0.0027 M KCl, 0.0101 M Na_2_HPO_4_, 0.0018 KH_2_PO_4_ was used as the basic electrolyte.

## Abbreviations

BSA: Bovine serum albumin
DPM: thiol derivative of dipyrromethene
EDC/NHS: 1-Ethyl-3-(3-dimethylaminopropyl) carbodiimide/N-hydroxysuccinimide
EIS: Electrochemical impedance spectroscopy
ELISA: Enzyme-linked immunosorbent assay
HA: Hemagglutinin
His_6_-H1 HA: Recombinant His-tagged hemagglutinin
MBT: 4-mercapto-1-butanol
OSWV: Osteryoung Square Wave Voltammetry
PBS: Phosphate buffer saline
SAM: Self-assembled monolayers
WHO: World Health Organization
